# Advanced pneumatic compression for treatment of lymphedema of the head and neck: a randomized wait-list controlled trial

**DOI:** 10.1007/s00520-020-05540-8

**Published:** 2020-06-02

**Authors:** Sheila H. Ridner, Mary S. Dietrich, Jie Deng, Sandra L. Ettema, Barbara Murphy

**Affiliations:** 1 Vanderbilt School of Nursing, Vanderbilt University School of Nursing, 461 21st Avenue South, Nashville, TN 37240 USA; 2 Vanderbilt School of Nursing, Vanderbilt University Medical Center, 1211 Medical Center Drive, Nashville, TN 37232 USA; 3 University of Pennsylvania School of Nursing, 418 Curie Boulevard, Philadelphia, PA 19104-4217 USA; 4 Southern Illinois University School of Medicine, P.O. Box 19620, Springfield, IL 62794-9620 USA; 5 Vanderbilt University Medical Center, 1211 Medical Center Drive, Nashville, TN 37232 USA

**Keywords:** Head and neck cancer, Lymphedema, Fibrosis, Pneumatic compression device

## Abstract

**Purpose:**

Lymphedema associated with head and neck cancer (HNC) therapy causes adverse clinical outcomes. Standard treatment includes professionally administered complete decongestive therapy (CDT). Cost and availability of trained therapists are known barriers to therapy. Advanced pneumatic compression devices (APCD) may address these issues. A randomized, wait-list controlled trial was undertaken to evaluate an APCD in post-treatment HNC patients with lymphedema.

**Material and methods:**

Eligible patients had completed treatment for HNC, were disease free, and had lymphedema at enrollment. Participants were randomized to wait-list lymphedema self-management (standard of care) or lymphedema self-management plus the use of the APCD bid. Safety (CTCAE V4.0) and feasibility were primary endpoints; secondary endpoints included efficacy measure by objective examination and patient reported outcomes (symptoms, quality of life, function), adherence barriers, and satisfaction. Assessments were conducted at baseline and weeks 4 and 8.

**Results:**

Forty-nine patients were enrolled (wait-list *n* = 25; intervention *n* = 24). In total, forty-three patients completed the study. No device-related Serious Adverse Events were reported. Most patients used the APCD once per day, instead of the prescribed twice per day, citing time related factors as barriers to use. APCD use was associated with significant improvement in perceived ability to control lymphedema (*p* = 0.003) and visible external swelling (front view *p* < 0.001, right view *p* = 0.004, left *p* = 0.005), as well as less reported pain.

**Conclusion:**

This trial supports the safety and feasibility of the APCD for the treatment of secondary lymphedema in head and neck cancer patients. In addition, preliminary data supports efficacy.

## Purpose

Head and neck cancer (HNC) and its therapy are associated with damage to lymphatic structures resulting in secondary lymphedema [1–5]. In one prospective trial, over 90% of HNC patients developed lymphedema at some time during the course of their treatment and early recovery [4]. Lymphedema manifestations vary depending on the involved site. External lymphedema of the face and neck is associated with decreased range of motion (ROM), abnormal posture, and musculoskeletal discomfort. Lymphedema involving internal structures such as the pharynx or larynx may result in airway compromise and/or dysphagia [2–4]. Lymphedema is often linked to psychological distress and decreased quality of life [6].

Mild lymphedema may be present at the time of head and neck cancer diagnosis [4]. Incidence increases after definitive or postoperative radiation therapy. A majority of patients develop moderate to severe lymphedema with associated symptom burden and/or altered function. Available data indicate that some patients develop late stage lymphedema, characterized by fibrofatty scar tissue [7]. Progressive soft tissue changes may be related to lymphedema associated chronic inflammation which in turn may lead to a self-perpetuating and worsening clinical presentation [8]. Thus, aggressive and early treatment of lymphedema is critical to optimize long-term patient outcomes.

Standard treatment for lymphedema is complete decongestive therapy with manual lymphatic drainage, education, compression, exercise, and skin care. Most HNC patients undergo brief professional therapy followed by self-care. While this approach may prove efficacious for some patients, lymphedema fails to respond adequately to standard therapies in others [9–13]. Many patients experience barriers to lymphedema care including cost or insurance obstacles, lack of certified and experienced lymphedema therapists, and self-limitations (e.g., physical or cognitive impairments). Thus, cost effective, home based treatment options for both primary and refractory lymphedema remains an unmet need. In response, Tactile Medical™ has developed a garment for the treatment of head and neck lymphedema utilizing The Flexitouch® System advanced pneumatic compression device (FT). The head and neck application of the Flexitouch system received 510(k) pre-market notification clearance from the FDA in August of 2016. The purpose of this study was to evaluate the feasibility and efficacy regarding the use of the FT in HNC survivors with lymphedema.

## Material and methods

### Design and participants

This was an open label, randomized, wait-list controlled study conducted at Vanderbilt University and Southern Illinois University School of Medicine. Patients were randomly assigned to receive usual care or FT, according to a sequence of computer-generated random numbers, with stratification by study site. The study was conducted in accordance with the ethical standards of the Helsinki Declaration and registered at ClinicalTrial.gov number NCT03332160. Institutional Review Board (IRB) and Scientific Review Committee approvals were obtained prior to patient recruitment. Eligible patients had completed cancer treatment for histologically proven HNC, recovered from acute treatment effects, and had no evidence of active disease. Patients had a clinical diagnosis of lymphedema in the head and neck region. They had either undergone lymphedema therapy or were unable to access therapy due to defined barriers such as lack of available clinical services or socioeconomic constraints (e.g., lack of insurance, lack of transportation). Additional inclusion criteria included age ≥ 18 years; and able and willing to participate in all aspects of the study; and to provide informed consent. Exclusion criteria included a documented history of: (1) uncontrolled hyperthyroidism or parathyroidism; (2) carotid sinus hypersensitivity syndrome; (3) symptomatic carotid artery disease, as manifested by a recent transient ischemic attack (within 30 days), ischemic stroke, or amaurosis fugax (monocular visual ischemic symptoms or blindness); (4) symptomatic bradycardia in the absence of a pacemaker; (5) internal jugular venous thrombosis, acute or within 3 months; (6) increased intracranial pressure or other contraindications to internal or external jugular venous compression; (7) acute radiation dermatitis, unhealed surgical scar, unhealed or open wound(s), surgical flap less than 6–8 week post-operative; (9) acute facial infection (e.g., facial or parotid gland abscess); (10) any condition in which increased venous and lymphatic return was undesirable (example: history of pulmonary edema or decompensated congestive heart failure within six (6) weeks of enrollment); (11) pregnancy or trying to become pregnant; and (12) interference with tracheostomy function by garment.

### Methods

Research team members were trained to conduct and document head and neck physical examinations by authors Ridner and Murphy. Eligible patients were consented, and then measured for garment size selection. No subjects were withdrawn due to poor garment fit. Baseline evaluation included a physical exam, endoscopy, completion of questionnaires, and bloodwork. After baseline evaluation, participants were randomized to either wait-list lymphedema self-management (standard of care) or lymphedema self-management plus the use of the FT twice daily for 8 weeks. Time allotted for use varied based upon size of garment and ranged from 23 to 45 min.

All patients received a self-care kit that included a diary, self-care checklist, and date and times of future study appointments. The intervention group received the FT and was instructed on use, including the timer that would record their actual time on the machine. All patients had follow-up visits at 1, 4, and 8 weeks during which they were assessed for adverse events and completion of study measures. For patients in the intervention group, study participation concluded at week 8. Patients assigned to the wait-list group could opt to continue on study for the purpose of using the FT. If they opted to do so, they (1) were provided with the FT for an 8-week treatment period; (2) given the same education prior to use as intervention group; and, (3) were seen subsequently for safety checks at 1, 4, and 8 weeks post-receipt of the FT.

### Data collection and instruments

Patients completed a demographic survey at baseline. Disease and treatment data were extracted from medical records.

### Safety and feasibility

Safety was evaluated using CTCAE V4.0 [14]. Severe or unexpected adverse events were reportable to the IRB. All patients completed a weekly self-care checklist. The date and time of use were recorded by the device. Those data were exported and analyzed for determining the frequency and duration (minutes) of use per day for patients assigned to the intervention group. A daily diary was completed to document treatment barriers. A six-item survey regarding perceived lymphedema control, management, and health was completed by intervention participants at the baseline, and end of study.

### Objective assessments

A head and neck physical exam was conducted by trained study personnel. External lymphedema and fibrosis were ascertained by touch and visual inspection. External grading was documented using the Head and Neck Lymphedema and Fibrosis Assessment criteria [15]. Using this tool, skin and soft tissue changes were typed as follows: A—involving skin only, B—reducible soft tissue swelling, C—firm, non-reducible swelling, and D—fibrosis without swelling. Types B, C, and D are then graded as mild, moderate, or severe. The site of soft tissue abnormalities was documented in a table format that includes left and right periorbital region, left and right cheeks, left and right neck, left and right supraclavicular region, and the submental area.

Endoscopic exams were performed by a trained, blinded Otolaryngology nurse practitioner. Internal lymphedema was scored using the Modified Patterson Scale [16]. A grade of normal, mild, moderate, or severe was documented for each site or space.

Digital photographs of the head and neck, each profile and facing forward, were taken and overlaid with a 30 segment grid. Each segment was rated yes/no by a blinded single rater for swelling. A composite score of percentage of grids with swelling was used as an indicator of swelling extent.

### Patient reported outcomes

#### Subjective assessments

Patients completed symptom assessment and quality of life measures at baseline, 4, and 8 weeks. The Lymphedema Symptom Intensity and Distress Survey-Head and Neck (LSIDS-HN) is a 48-item tool that captured symptom intensity and distress, both independently rated on a scale of 1 (slight) to 5 (severe), yielding a total potential symptom burden score of 10 [3, 17]. The Vanderbilt Head and Neck Symptom Survey plus General Symptom Survey version 2.0 (VHNSS v2.0 plus GSS), a 61-item tool, was used to assess the prevalence and severity of HNC treatment-related symptoms and their functional impact [18]. A 5-item Linear Analog Self-Assessment was used to evaluate quality of life (QOL) [19].

#### Function

Cervical range of motion (CROM) were taken using the cervical and shoulder range of motion instrument [20]. Jaw range of motion (ROM) was documented using the TheraBite Jaw ROM Scale [21]. Trismus grading criteria from the CTCAE v4.0 was recorded [14]. Patients completed the Neck Disability Index (NDI) to assess components of daily life that may be affected by neck pain and dysfunction [22], and the self-report Voice Handicap Index (VHI) garnered data regarding voice disorders [23].

#### Correlative studies

Blood samples were obtained at baseline and at the 8-week visit for the following inflammatory markers: IFNg, TNF-α, TGF-β1, IL-1b, and IL-6.

#### Analysis

Descriptive statistics were used to summarize the demographic, clinical history, and outcome variables in the study. Due to skewness of many of the data distributions, median (IQR) was used for describing the continuous variables. Characteristics of the patients assigned to the separate study arms were compared using Mann-Whitney and Chi-Square tests. Given the preliminary nature of this work, small sample, and considerable variability among the patient scores at baseline, initially the change in the score for each outcome measure was calculated for each patient. Differences between the groups from baseline to the end of the 8-week study period were then conducted using generalized linear regressions that included the patient’s respective baseline values for the outcome variable being analyzed. Inclusion of the baseline values allowed us to control for potential differences between the groups at initial time of assessment and focus on differences between the groups in the amount of change from baseline. An alpha of 0.05 was used for evaluation of statistical significance, and no corrections for multiple tests were used in this preliminary study. Feasibility, adherence, and safety of the FT were the primary outcomes, with efficacy included to generate initial estimates of effect for larger future trials. The adjusted *beta* coefficient for the study group effect generated by each regression was transformed to the Cohen’s *d* effect statistic for ease of interpretation of the study effects on patient outcomes.

## Results

### Patient characteristics

**Fig. 1:**
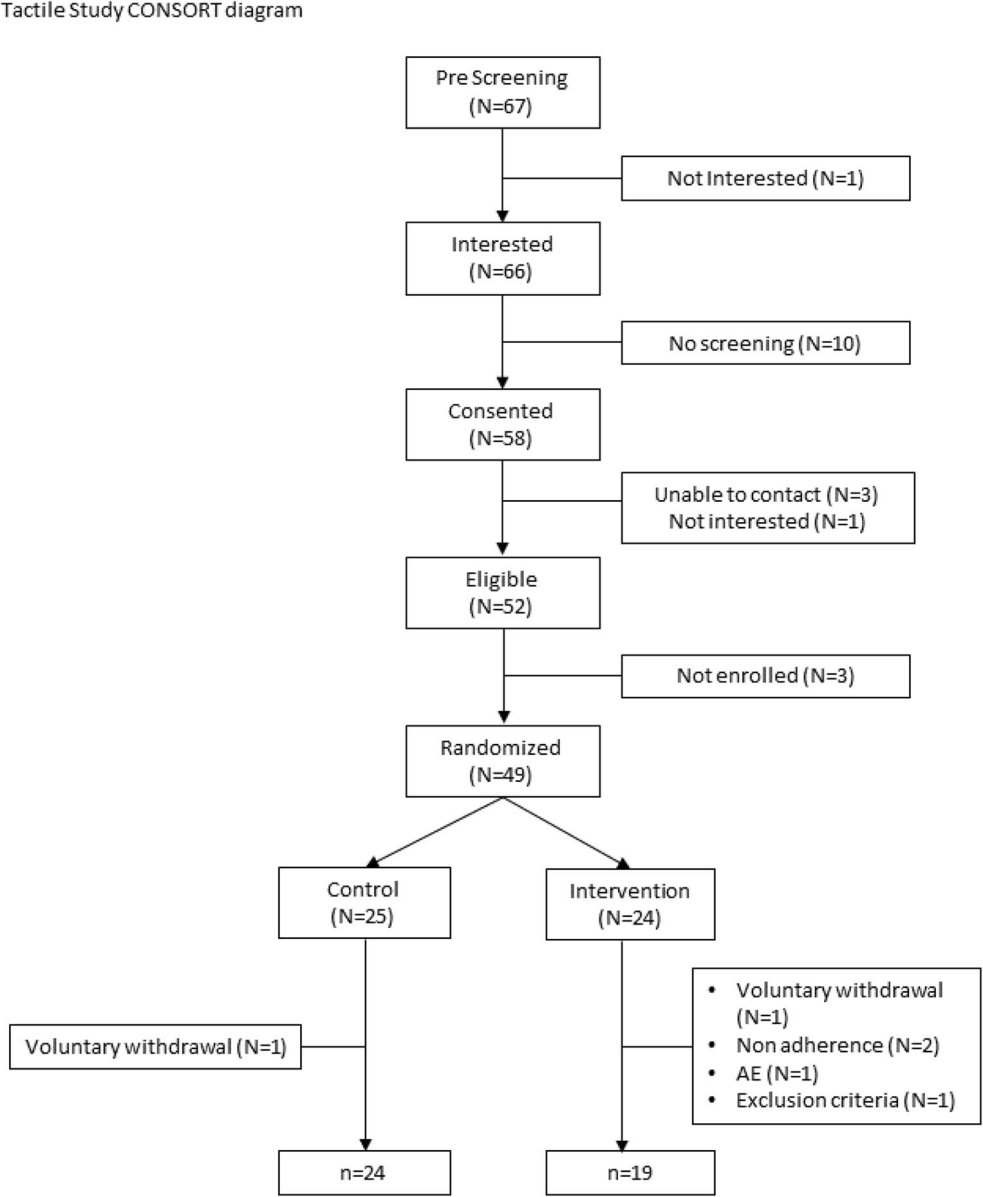
Tactile study CONSORT diagram

Twenty-five patients were randomly assigned to the control group, and 24 to the intervention group (Fig. 1). One patient withdrew from the control group immediately after learning he was wait-listed, leaving 24 in the control group. Five withdrew from the intervention group: 2 PI withdrawals for failing to use the device for 7 consecutive days, 1 became ineligible, 1 self-withdrew, and 1 non-device related AE, leaving 19 in the intervention group. The final sample (*N* = 43) consisted primarily of non-Hispanic white (97.7%) males (81.4%) in their early 60’s (median = 62.2) who had been diagnosed with lymphedema approximately 5 months prior to enrollment (median = 5.2, min = < 1 month, max = 37 months). Characteristics were very similar for patients in the two groups (see Table 1).

**Table 1:** Demographic and health characteristics

Characteristic^a^	Overall (*N* = 43)	Control (*n* = 24)	Intervention (*n* = 19)	*p* value
	Median [IQR]	Median [IQR]	Median [IQR]	
Age	62.2 [56–68]	62.8 [57–69]	61.1 [54–68]	0.359
Gender	N (%)	N (%)	N (%)	0.714
Female	8 (18.6)	4 (16.7)	4 (21.1)	
Male	35 (81.4)	20 (83.3)	15 (78.9)	
Race				0.255
Multiple	1 (2.3)	0 (0.0)	1 (5.3)	
White	42 (97.7)	24 (100.0)	18 (94.7)	
Education				0.115
<= High school	16 (37.2)	10 (41.7)	6 (31.6)	
College	21 (48.8)	13 (54.2)	8 (42.1)	
Advanced degree	6 (14.0)	1 (4.2)	5 (26.3)	
Marital Status				0.564
Married/living with partner	37 (86.0)	20 (83.3)	17 (89.5)	
Other	6 (14.0)	4 (16.7)	2 (10.5)	
Residence				0.385
City	15 (34.9)	8 (33.3)	7 (36.8)	
Country	18 (41.9)	12 (50.0)	6 (31.6)	
Suburb	10 (23.3)	4 (16.7)	6 (31.6)	
BMI group at enrollment	N (%)	N (%)	N (%)	0.676
Underweight	3 (6.3)	1 (4.2)	2 (8.3)	
Normal/healthy weight	8 (16.7)	5 (20.8)	3 (12.5)	
Overweight	18 (37.5)	10 (41.7)	8 (33.3)	
Obese	19 (39.6)	8 (33.3)	11 (45.8)	
Smoking history/current	28 (65.1)	16 (66.7)	12 (63.2)	0.811
Alcohol history/current	32 (74.4)	18 (75.0)	14 (73.7)	0.922
Trach at Enrollment	6 (14.3)	4 (17.4)	2 (10.5)	0.527
PEG at Enrollment	12 (28.6)	6 (26.1)	6 (31.6)	0.695
	Median [IQR]	Median [IQR]	Median [IQR]	
Months since lymphedema diagnosis	5.2 [1–14]	5.3 [1–10]	5.2 [1–18]	0.608
Months since initial lymphedema treatment	4.6 [2–12]	4.6 [2–9]	4.5 [2–22]	0.746

^a^All 43 participants were Non-Hispanic

### Safety and feasibility

There were four serious adverse events (grade 3 or 4) all unrelated to device use. Those events included one of each of the following: cellulitis, stroke, hyponatremia, and death. The most common adverse events included erythema, edema, ecchymosis, tenderness, numbness, and hard lumps. None were severe; all were temporary and resolved without medical attention.

Data captured by the diary and FT indicated a low rate of user error: one patient did not plug in a connector. Data demonstrate overall lack of compliance with the prescribed regimen. During weeks 1 and 2, only 26% (5 of 19) met the prescribed use criteria; 47% (9 of 19) met those criteria 5 of the 7 days during the first 2 weeks. Those rates stayed generally stable through week 7. During week 8, only 21% (4 of 19) used the FT as prescribed for at least 5 days, and only 1 person used it twice a day, every day, during that week. Two barriers to use were identified. Time related factors included family issues (*n* = 3), work (n = 3), and travel (*n* = 2). Discomfort related factors included pressure/metal (*n* = 1), noise (*n* = 1), garment fit (*n* = 1), and “not feeling well” (*n* = 1).

### Patient reported outcomes

Patients in the intervention group reported improvement in perceived ability to control lymphedema (baseline: 5/19, 26% good or excellent; 8 weeks: 16/19, 84% good or excellent, *p* = 0.003).

Relative to patients in the control group, at 8-weeks patients using the FT had statistically significant reductions in the reported severity of soft tissue (*p* = 0.008, *d* = − 0.86) and neurological symptom (*p* = 0.047, *d* = − 0.60) clusters on the LSIDS-HN. Examples of soft tissue symptoms include “heaviness,” “tightness,” and “swelling”; neurological symptoms included “tingling” and sensations of “pins and needles”. While not statistically significant, the next strongest effects were on reported oral symptoms (*p* = 0.099, *d* = − 0.53; e.g., “difficulty swallowing,” “difficulty moving tongue”) and impact of symptoms on activity (*p* = 0.080, *d* = − 0.58; e.g., “difficulty bending,” “decreased social activity”) (see Table 2).

**Table 2:** LSIDS max score summaries^a^

LSIDS Max Score	Baseline	Change	Cohen’s *d*	*p* value
	Median [IQR]	Median [IQR]		
Soft tissue			− 0.86	0.004
Control	6.0 [3, 7]	0.0 [0, + 2]		
Intervention	5.0 [3, 6]	− 2.0 [− 2, 0]		
Neurological			− 0.60	0.047
Control	5.5 [2, 6]	0.0 [0, + 2]		
Intervention	2.0 [0, 6]	0.0 [− 2, 0]		
Oral			− 0.53	0.099
Control	6.0 [4, 8]	0.0 [− 1, + 2]		
Intervention	4.0 [0, 7]	0.0 [− 2, 0]		
Biobehavioral			− 0.30	0.350
Control	6.0 [4, 8]	0.0 [− 2, + 1]		
Intervention	4.0 [0, 6]	0.0 [− 2, + 1]		
Resources			< 0.01	0.988
Control	0.0 [0, 0]	0.0 [0, 0]		
Intervention	0.0 [0, 0]	0.0 [0, 0]		
Sexuality			− 0.13	0.674
Control	0.0 [0, 5]	0.0 [0, + 2]		
Intervention	0.0 [0, 4]	0.0 [0, + 1]		
Activity			− 0.58	0.080
Control	7.5 [5, 8]	0.0 [− 3, + 1]		
Intervention	2.0 [0, 6]	0.0 [− 3, 0]		
Function			− 0.21	0.479
Control	6.0 [4, 8]	0.0 [− 1, + 2]		
Intervention	4.0 [0, 6]	0.0 [− 1, + 1]		

^a^Control *N* = 24, Intervention *N* = 19; possible range of values for each scale was 0–10

Cohen’s *d* and p value are for differences in the amount of change between the groups (controlling for baseline)

Relative to patients in the control group, patients using the FT had statistically significant improvement in swallowing solids (*p* = 0.016) and mucous related symptoms (*p* = 0.050, *d* = − 0.80 and − 0.57 respectively) on the VHNSS-GSS. Furthermore, patients in the control group reported an increase in general pain over the study period, while patients in the intervention group reported essentially the same level as at baseline (*p* = 0.008, *d* = − 0.89). No other statistically significant differences in changes in the VHNSS-GSS symptoms were noted, with the next strongest effect demonstrated for swallowing liquids (*d* = − 0.49, *p* = 0.099).

After controlling for baseline differences between the groups, no statistically significant difference in changes in the NDI, VHI, or QOL outcomes were observed.

### Objective assessments

**Fig. 2:**
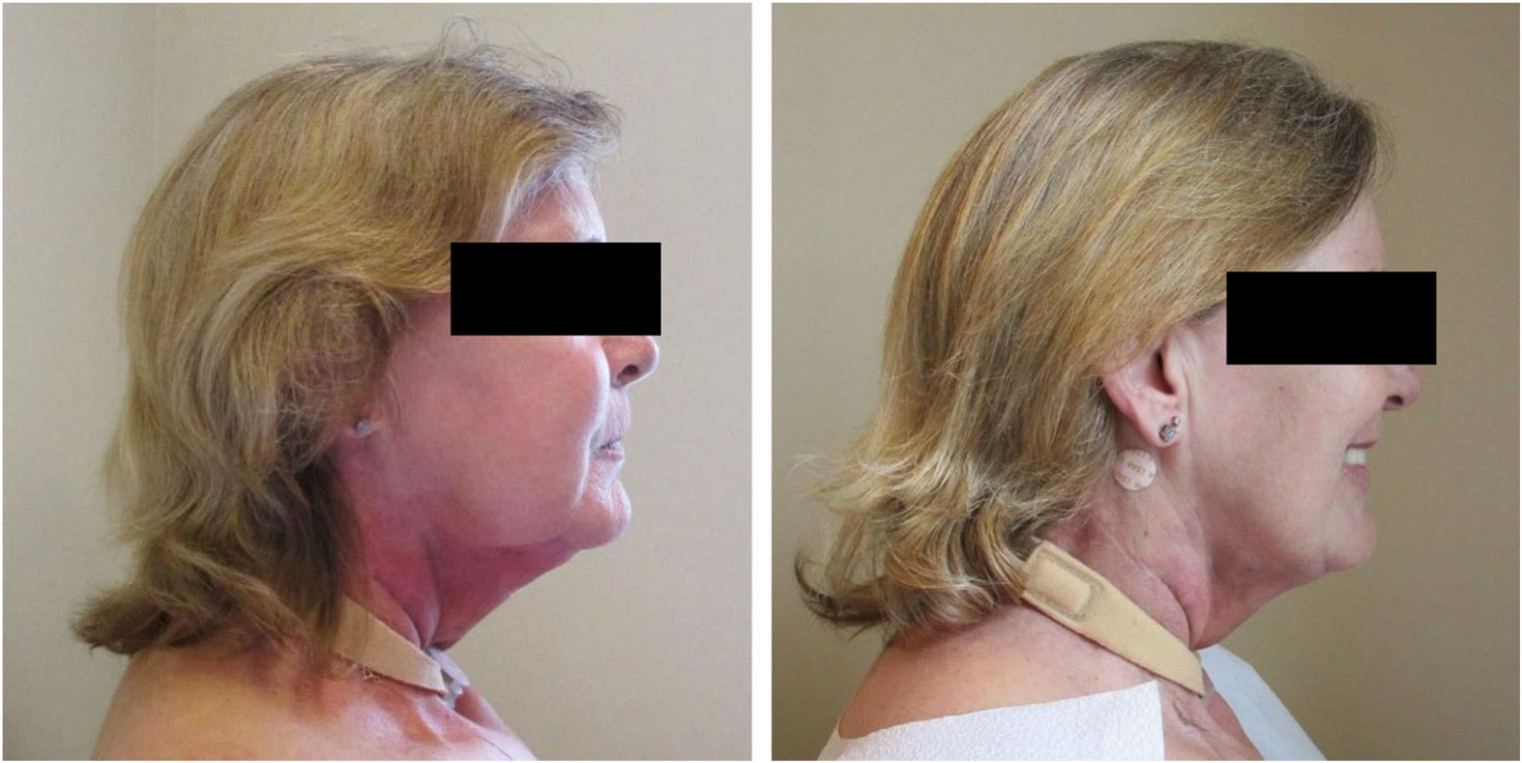
An exemplar intervention subject before randomization and after last 8-week treatment (left to right)

Photos of the patients in the intervention group demonstrated a greater decrease in the percentage of grids with observable swelling at 8-weeks versus the control group photos (see an exemplar set of photos in Fig. 2). Front view reduction was a median 24% (vs. + 5% control, *p* < 0.001, *d* = − 1.26), right view reduction a median 22% (vs. − 7% control, *p* = 0.004, *d* = − 0.96), and left view a median 17% (vs. − 4% control, *p* = 0.005 *d* = − 0.84).

Physical examination revealed a change in both the number of swollen sites and the severity of swelling from baseline to end of treatment in favor of the FT group. The difference between groups did not reach statistical significance (number of sites: *p* = 0.209, *d* = − 0.38; total severity: *p* = 0.094, *d* = − 0.50). Differences in internal swelling via endoscopic evaluation were not statistically significant between groups in either the change in the percentage of visible sites with swelling (*p* = 0.961, *d* = 0.01), or in severity of the swelling (*p* = 0.948, *d* = 0.02).

### Function

No statistically significant differences in the amount of change from baseline to 8 weeks in the function measures were observed between groups. The largest effect was observed on the maximal inter-incisal distance (*d* = 0.32, *p* = 0.312). The largest effect difference on the CROM measures was on the right lateral flexion (*d* = 0.20, *p* = 0.435); effect difference on the VHI Functional score was − 0.15 (Cohen’s *d*, *p* = 0.615).

### Markers of inflammation

No statistically significant differences between the two groups of patients were observed in inflammatory biomarkers levels at 8 weeks (*p* > 0.10).

## Conclusion

The FT was safe and well tolerated in HNC survivors experiencing lymphedema. No SAE’s related to home use were reported. Patients were able to master the utilization of the FT without difficulty. User error was low, indicating ease of use and adequacy of patient education.

Once daily dosing is the standard recommendation for arm and leg Flexitouch systems. Given the complexity and clinical impact of head and neck lymphedema, the feasibility of a more aggressive, twice daily treatment regimen was tested. Adherence to the twice daily regimen was low. This result is unsurprising as patients who were compliant with twice daily treatments had available time to spend up to 1.5 h daily using their device. Time constraints were most commonly cited for non-adherence. Conversely, the data demonstrated that a once daily regimen was feasible. Thus, further studies should investigate a once daily treatment regimen.

Lymphedema is a chronic process requiring ongoing self-management. Thus, “perceived ability to self-manage” is one of the critical outcomes for lymphedema therapy. The results indicate that the FT significantly enhanced patient perception regarding their ability to control their lymphedema, potentially reducing patient distress. Empowering patients to manage their lymphedema and its associated symptoms may also result in long-term cost savings by decreasing the need for professional therapy sessions and mitigating long-term adverse effects.

Although the sample size in this study was small, there was significant improvement in lymphedema associated symptoms. The LSIDS is a tool that was developed to capture the unique and bothersome symptoms experienced by lymphedema patients. The soft tissue subscale includes items that capture altered sensation such as heaviness and tightness. Use of the FT significantly decreased soft tissue symptoms. Although the specific mechanism that underlies these symptoms has not been clearly elucidated, it may be hypothesized that peripheral mechanoreceptors in the soft tissues are activated within lymphedematous tissues and when adequately treated activation ceases resulting in decreased symptom burden. Similarly, use of the FT decreased symptoms such as numbness, and tingling or “pins and needles” sensation that were captured on the neurological subscale. These types of dysesthesias are usually the result of peripheral nerve damage, pressure on nerves, or lack of blood supply to the nerves. The FT may decrease symptomatology by decreasing pressure on nerves and improving blood supply to affected tissues. Further studies to explore the mechanism underlying sensory symptoms are warranted.

It is often held that patients with lymphedema do not experience significant pain; results of this study counter the prevailing wisdom. Furthermore, use of the FT was associated with stabilization of pain while patients in the control group experience worsening pain over time. Symptoms not expected to improve (e.g., taste, tooth sensitivity) with a decrease in lymphedema demonstrated no clinically significant difference between groups.

In addition to altered sensation, lymphedema underlies some of the long-term soft tissue symptoms experienced by HNC patients such as heaviness and is associated with substantial function loss. Swallow impairment is one of the most significant long-term soft tissue toxicities associated with lymphedema. It may be associated with acute episodes of aspiration pneumonia and chronic pulmonary fibrosis, dietary adaptations with associated micro and macronutrient deficiencies, and long-term requirement of a feeding tube. It has long been questioned as to whether lymphedema therapy may improve swallow function. The results of this study demonstrated that treatment of lymphedema, through use of the FT, improved patient reported swallow function. If this result is confirmed, then the FT may become an integral component of swallow therapy for post-treatment HNC patients with lymphedema associated dysphagia.

We used several objective measures including a newly developed clinician-report measure of external lymphedema, endoscopic evaluation of the soft tissues and spaces in the pharynx and larynx, a cervical range of motion device, measurement of interincisal distance, scoring of digital photographs, and cytokine assessment. Administration of these measures was feasible; however, the sample size was insufficient to determine the ability of some of these tools to capture lymphedema associated physiological or anatomical changes over time using the FT. Additionally, we did not exclude patients who had been biologics or anti-inflammatory medications, which may have impacted our findings. The statistically significant digital photograph finding in favor of the intervention group, however, is supported by a recent study that complied facial composite measurement scores using near infrared fluorescence lymphatic imaging [24]. These scores demonstrated that after 2 weeks of pneumatic compression therapy in the head and neck region, in 75% of the patients experienced disappearance of or reduced dermal backflow. Further investigation of these measures in larger studies is needed to determine the utility for measuring treatment outcomes of lymphedema therapy.

## Conclusions

Safety and feasibility outcomes support the use of the FT for the treatment of lymphedema in HNC patients secondary to cancer and its treatment. Adherence findings support daily vs. twice daily dosing. Symptom outcomes provide evidence for promising preliminary efficacy of the device. Further research in a larger RCT is indicated.
